# A DNA nanoflowers-based microneedle patch for transdermal gene and photodynamic therapy against melanoma

**DOI:** 10.1186/s12951-026-04095-w

**Published:** 2026-02-27

**Authors:** Yuchen Qi, Zhe Wang, Yaguang Wu, Qianqian Wu, Chong Li, Hua Yu, Tian Zeng, Yunlong Wang, Jianjun Li, Hang Qian, Xiang Zhao

**Affiliations:** 1 Department of Oncology, Southwest Hospital, Third Military Medical University (Army Medical University), Chongqing, 400038 China; 2 Department of Dermatology, Southwest Hospital, Third Military Medical University (Army Medical University), Chongqing, 400038 China; 3 Department of Oncology, Dazu Hospital of Chongqing Medical University, Chongqing, 402360 China; 4 Department of General Surgery, Hospital of Chengdu University of Traditional Chinese Medicine, Chengdu, 610072 China; 5 Department of Respiration and Critical Care Medicine, Xinqiao Hospital of Third Military Medical University (Army Medical University, 183 Xinqiao Street, Chongqing, 400037 China

**Keywords:** DNA nanoflower, Nanoarchitecture, Microneedle, Melanoma, Photodynamic therapy

## Abstract

DNA nanostructures show great potential as drug carriers in biomedicine, however, biological barriers and stability issues limit their applications, particularly for aggressive melanoma shielded by the skin barrier. This study presents a hyaluronic acid-based microneedle patch integrated with DNA nanoflowers to effectively penetrate the skin barrier. The DNA nanoflowers (Z/H@DFs) are self-assembled from PD-L1 antisense oligonucleotide (ASO), AS1411 G-quadruplex units, Hemin, and ZnPc. In a B16F10 melanoma mouse model, the Z/H@DFs-based microneedle patches (Z/H@DFMNs) significantly inhibited tumor growth. Mechanistically, Z/H@DFMNs patch relieved hypoxia, promoted the photodynamic therapy effect, and activated anti-tumor immunity via the suppression of PD-L1 expression. The proposed synergistic photo-immunotherapy strategy could realize 3-fold enhancement in tumor infiltrating CD8^+^ T cells and over 2-fold increase in serum IFN-γ and TNF-α levels. The Z/H@DFMNs patch is a potential solution for melanoma immunome and other skin disorders.

**Graphical Abstract:**

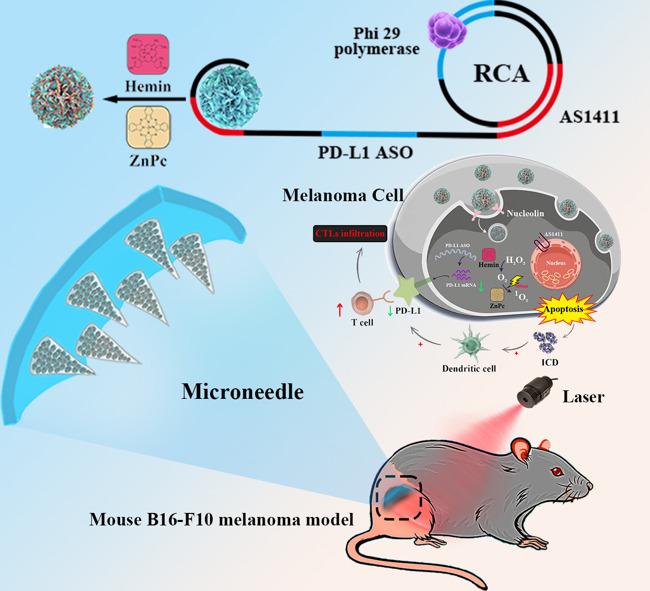

**Supplementary Information:**

The online version contains supplementary material available at 10.1186/s12951-026-04095-w.

## Introduction

Melanoma is a malignant skin cancer with a mortality rate surpassing that of other skin cancers due to its invasive tendencies, susceptibility to early metastasis, and drug resistance [1]. Even if it is suitable for surgical and chemotherapy/radiation therapy, they suffer from some defects like a large amount of skin damage, systemic toxicity, drug resistance, and metastasis [2]. Emerging nanomedicine offers promising strategies to tackle melanoma through targeted drug delivery and enhanced therapeutic efficacy. It can improve the bioavailability and circulation time of drugs and can also be engineered to overcome biological barriers, such as the skin barrier, to ensure effective delivery of therapeutics to the tumor site. However, challenges for nanomedicine in tackling melanoma include achieving specific targeting of tumor cells, minimizing off-target effects, reducing systemic toxicity, addressing tumor heterogeneity, and overcoming regulatory and manufacturing obstacles. Therefore, it is advantageous to explore more powerful therapeutic strategies for modifying the tumor microenvironment, improving the effectiveness of immunotherapy, and creating safe, highly efficient treatment methods.

DNA nanostructures hold promise for biomedical applications due to their unique properties such as programmability, biocompatibility, and self-assembly capabilities [3]. To date, they have demonstrated significant potential as versatile tools for drug delivery [4], gene therapy [5], biosensing [6], and tissue engineering [7]. DNA nanoflowers (DFs) are self-assembled nanostructures composed of DNA strands that form flower-like shapes. Their unique properties, including biocompatibility, programmability, and hierarchical nanoarchitecture [8], make DFs a promising platform for various applications in medicine and biotechnology [9]. *Wiraja et al.* designed a series of framework nucleic acids (FNAs) with varying shapes and sizes and found that FNAs with smaller nanostructures less than 75 nm can effectively penetrate the dermis layer. An impressive penetration depth of up to 350 μm from the skin periphery was achieved with a 17 nm tetrahedral FNAs [10]. In a study by *Lin* *et al.*, responsive tetrahedral FNAs carrying a miRNA-31 inhibitor demonstrated superior skin penetration ability, leading to significant anti-aging effects [11]. Another study by *Shen et al*. involved the construction of tetrahedral FNAs for the transdermal delivery of CpG oligodeoxynucleotide, doxorubicin, and PD-L1 inhibitors, resulting in enhanced anti-tumor performance for malignant melanoma [12]. Compared to traditional nanocarriers such as liposomes, DFs exhibit superior structural stability and programmability, while their highly branched, dense architecture offers a larger payload capacity and more functionalization sites than conventional DNA nanostructures like tetrahedral FNAs [9]. While DFs exhibit robust potential for drug delivery, critical knowledge gaps persist in understanding their dynamic transport mechanisms and active targeting strategies. Further investigations into DFs-skin matrix interactions and surface engineering approaches are essential for optimizing these nanomaterials’ clinical translatability.

The appeal of transdermal drug delivery lies in its ability to circumvent hepatic first-pass metabolism and gastrointestinal degradation, while also minimizing systemic side effects by facilitating localized drug delivery. Nevertheless, traditional transdermal drug delivery methods are hindered by poor skin permeability, limiting drug efficacy. In response, microneedles (MNs) have been developed as a novel therapeutic approach for dermatological disorders [13]. Unlike conventional transdermal drug delivery systems, which primarily rely on passive diffusion mechanisms, MNs possess the unique capability to penetrate the stratum corneum, the outermost layer of the skin. By creating a multitude of microscopic channels, or micropores, in this otherwise impermeable barrier, MNs facilitate a more efficient pathway for drug transport. It allows for uniform and sustained diffusion of therapeutic agents into the deeper layers of the skin, particularly through the spinous and basal cell layers of the epidermis [14]. Hyaluronic acid (HA)-based MNs address this by leveraging their biocompatibility to enhance tissue penetration and localized drug release.

**Scheme 1:**
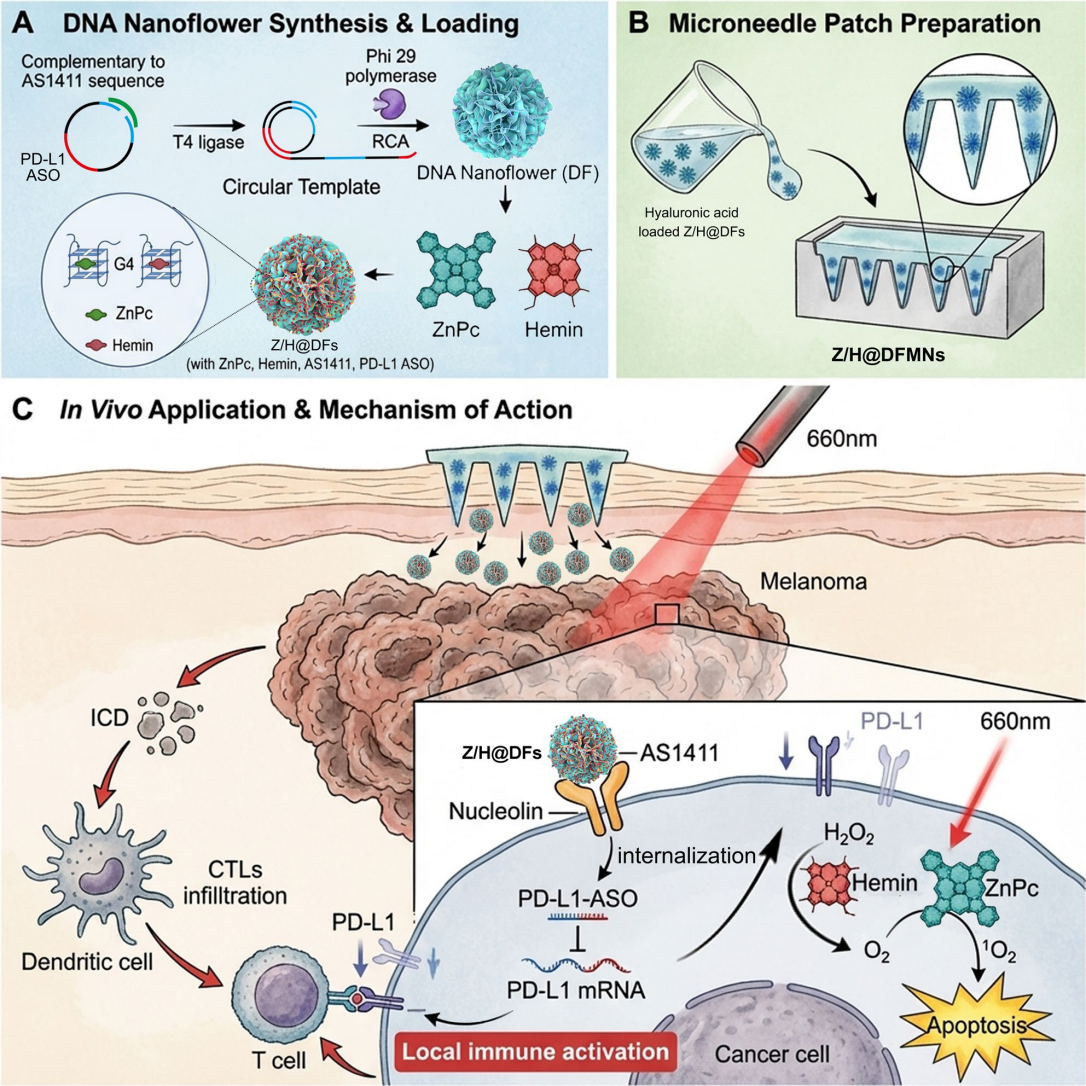
Schematic illustration of a Z/H@DFs-based microneedle (Z/H@DFMNs) patch with photo-immunotherapy capabilities for combating melanoma. The Z/H@DFs contains AS1411 and PD-L1 ASO units and carries ZnPc and Hemin via the G-quadruplex structures of AS1411. A Z/H@DFMNs patch is then fabricated for the treatment of melanoma. Upon insertion into the tumor tissues, the Z/H@DFMNs dissolves and slowly releases Z/H@DFs for synergistic treatment of PDT and PD-L1 ASO-based gene therapy against melanoma.

In this study, we propose to develop a DFs-based microneedle patch for transdermal gene and PDT against melanoma. Specifically, the DFs are designed to consist of AS1411 aptamer and PD-L1 antisense oligonucleotide (ASO) sequences (Scheme 1). The fabrication process entailed the utilization of rolling circle amplification (RCA) reaction. Notably, AS1411 exhibits tumor-targeting properties that facilitate cellular internalization, enabling the delivery of ZnPc and Hemin carried within its G-quadruplex structure into target tumor cells [15]. G-quadruplex enhances Hemin-catalyzed hydrogen peroxide decomposition to alleviate hypoxia, and promotes reactive oxygen species (ROS) generation by ZnPc under light irradiation, thereby enhancing phototherapeutic efficacy. The PD-L1 ASO was specifically engineered as a gene therapy modality to target and degrade PD-L1 mRNA in murine models, thereby suppressing PD-L1 expression within tumor cells, guiding immune checkpoint blockade (ICB), and synergizing with PDT to achieve enhanced photo-immunotherapy against melanoma [16]. Moreover, the high cargo capacity and stability of DFs overcome limitations associated with conventional nanocarriers in MNs, ensuring efficient co-delivery and sustained release of diverse therapeutic agents. Subsequently, the DFs are integrated into HA-based MNs systems (DFMN). When inserted into the tumor site, the DFMN tip dissolves in the interstitial fluid, liberating the encapsulated DFs and facilitating dispersion throughout the tumor tissue [17]. *In* *vitro* and *in vivo* experimental results demonstrated the potent efficacy of DFs combined with the MNs technique in combating melanoma by reshaping the immune microenvironment of cold tumors. The proposed strategy opens up more possibilities for the clinical management of malignancy.

## Results and discussion

### Synthesis and characterization of DFs

**Fig. 1:**
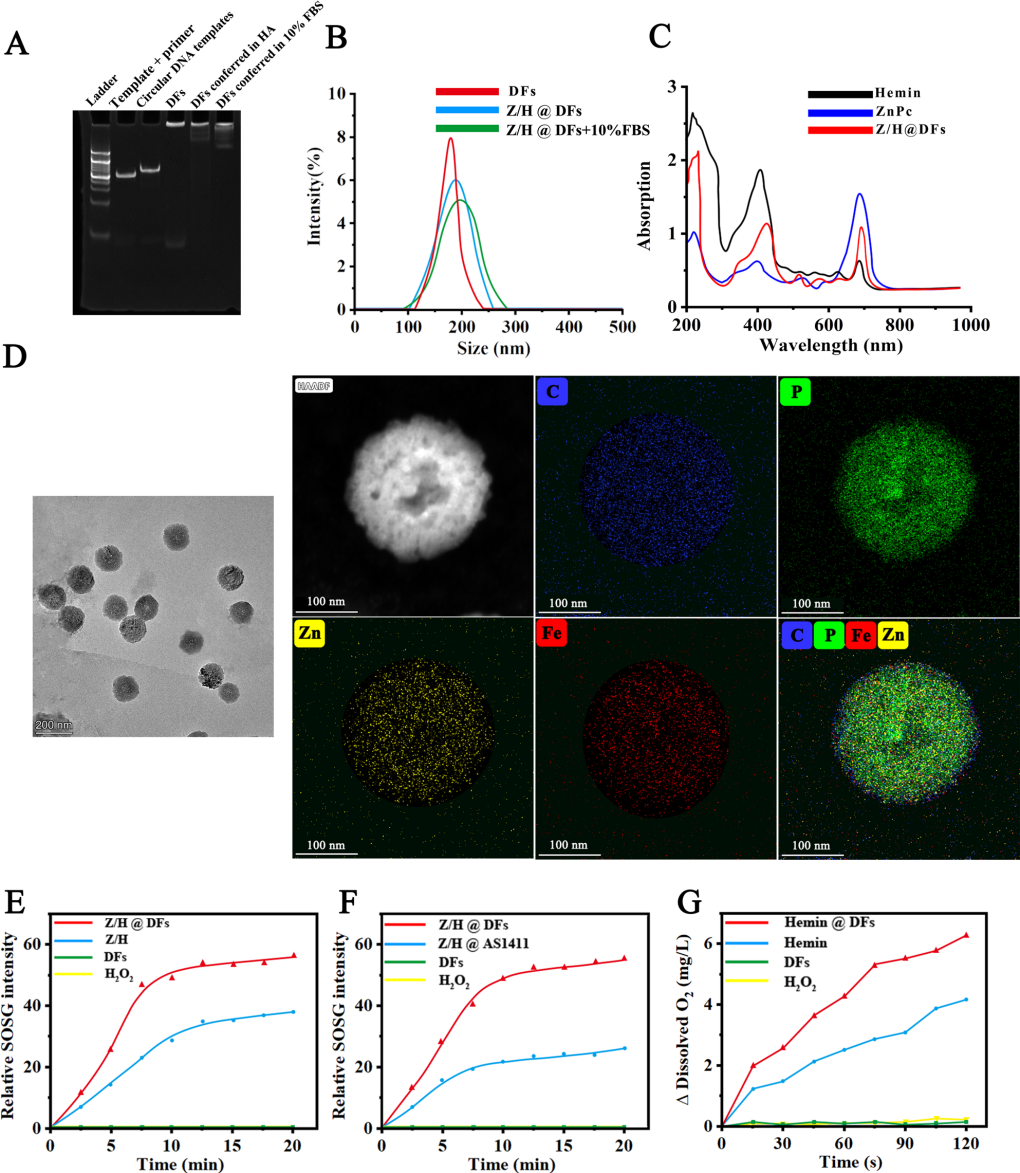
Synthesis and characterization of DFs and Z/H@DFs. **A** PAGE analysis of template-primers complex, circular DNA templates, RCA products and RCA products conferred in HA/10% FBS for 24 h. **B** DLS of DF, Z/H@DFs and Z/H@DFs+10% FBS. **C** UV-Vis spectra of Hemin, ZnPc and Z/H@DFs. **D**. Transmission electron microscope imaging and the elemental mapping image of Z/H@DFs. **E** The ability of different reactive groups to produce ^1^O_2_. **F** The ability of different reactive groups to produce ^1^O_2_ after incubation with 10% FBS. Z/H@AS1411 refers to ZnPc and Hemin-loaded AS1411 aptamers. **G** The ability of different reactive groups to produce O_2_

The synthesis of DFs particles followed a standardized procedure, which included the ligation of circular DNA templates by T4 ligase and subsequent rolling circle amplification [16]. The templates was customized to incorporate the AS1411 aptamer and the PD-L1 ASO functional units for specific functionalization within the resulting DFs. As shown in Fig. 1A, it can be observed that compared to the distinct bands formed by the linear template-primer complex and circular DNA template on the gel, the as-prepared DFs were trapped in the gel well due to their larger size and potential aggregation. Additionally, it was discovered that the majority of the DFs could retain their integrity when exposed to HA or 10% fetal bovine serum (FBS) for 24 h (Fig. 1A, and Fig. S1). The size of DFs was determined using dynamic light scattering (DLS). Naked DFs displayed a hydrodynamic size of 178.6 nm (Fig. 1B). After the incorporation of ZnPc and Hemin, the hydrodynamic sizes increased to 193.2 nm in PBS and 203.6 nm in 10% FBS. Further UV-vis spectroscopic analysis demonstrated the successful loading of ZnPc and Hemin into DFs (Z/F@DFs) (Fig. 1C). The peaks at 404 nm and 680 nm indicate the presence of Hemin and ZnPc, respectively. The transmission electron microscope (TEM) imaging confirmed the successful synthesis of DFs, which exhibited a uniform spherical morphology. Image-based counting analysis showed that DFs had an average diameter of 181 nm, which matched well with the DLS result. Elemental mapping results indicated the presence of Fe and Zn corresponding to the Hemin and ZnPc payloads in Z/H@DFs (Fig. 1D). These data collectively suggest that naked DFs and Z/H@DFs were successfully constructed. This synthesis highlights the programmability of DNA nanostructures, particularly the multifunctional role of the AS1411-derived G4 structures. Beyond serving as a tumor-targeting ligand, the G4 acts as a multi-functional platform: it disperses ZnPc to prevent aggregation and enhance its photosensitizing efficiency, while simultaneously providing binding sites for Hemin to form peroxidase-mimicking DNAzymes. From a materials science perspective, this elegant integration leverages the hierarchical architecture of DFs to achieve high payload capacity and stability, surpassing traditional carriers like liposomes or simpler DNA frameworks.

### In-situ oxygenation by ZnPc/Hemin-DFs for enhanced PDT

The efficacy of PDT is often limited by insufficient oxygen supply, along with challenges such as low photosensitizer solubility and limited energy conversion efficiency [18]. To address these issues, we strategically integrated Hemin and ZnPc via a G‑quadruplex structure. On one hand, this configuration significantly enhances the peroxidase-like activity of Hemin, helping to alleviate hypoxia in the tumor microenvironment. On the other hand, the AS1411 G‑quadruplex structure could improve the photosensitizing activity of ZnPc, facilitating efficient generation of ROS [19]. We thus examined the ROS generation behaviors of Z/H@DFs. Z/H@DFs exhibited much enhanced ROS production efficiency compared with Z/H (Free ZnPc with Hemin mixture), naked DFs, and H_2_O_2_ groups under laser irradiation (Fig. 1E). Moreover, our observations indicate that Z/H@DFs retain their robust ROS production capability even in the presence of serum (Fig. 1F). As expected, Z/H@DFs facilitates the rapid decomposition of H_2_O_2_ to yield O_2_, while DFs without Hemin and ZnPc loading did not exhibit obivous catalytic activity (Fig. 1G). It is reasonable to infer that DFs might have the potential to alleviate intratumoral hypoxia by catalyzing the decomposition of endogenous H_2_O_2_ abundant in the tumor microenvironment (TME) to generate O_2_ *in situ*. This process thereby enhances oxygen supply, counteracts TME-mediated immunosuppression, and boosts PDT efficiency [20]. Specifically, the peroxidase-like activity of Hemin efficiently catalyzes the decomposition of endogenous H₂O₂ in the TME into O₂, thereby directly increasing the local oxygen concentration available for PDT, leading to a measurable increase in ROS generation under laser irradiation. Consequently, the improved oxygen supply and ROS production collectively contribute to the enhanced PDT efficacy observed with Z/H@DFs.

### Cellular uptake and anticancer effects of Z/H@DFs

**Fig. 2:**
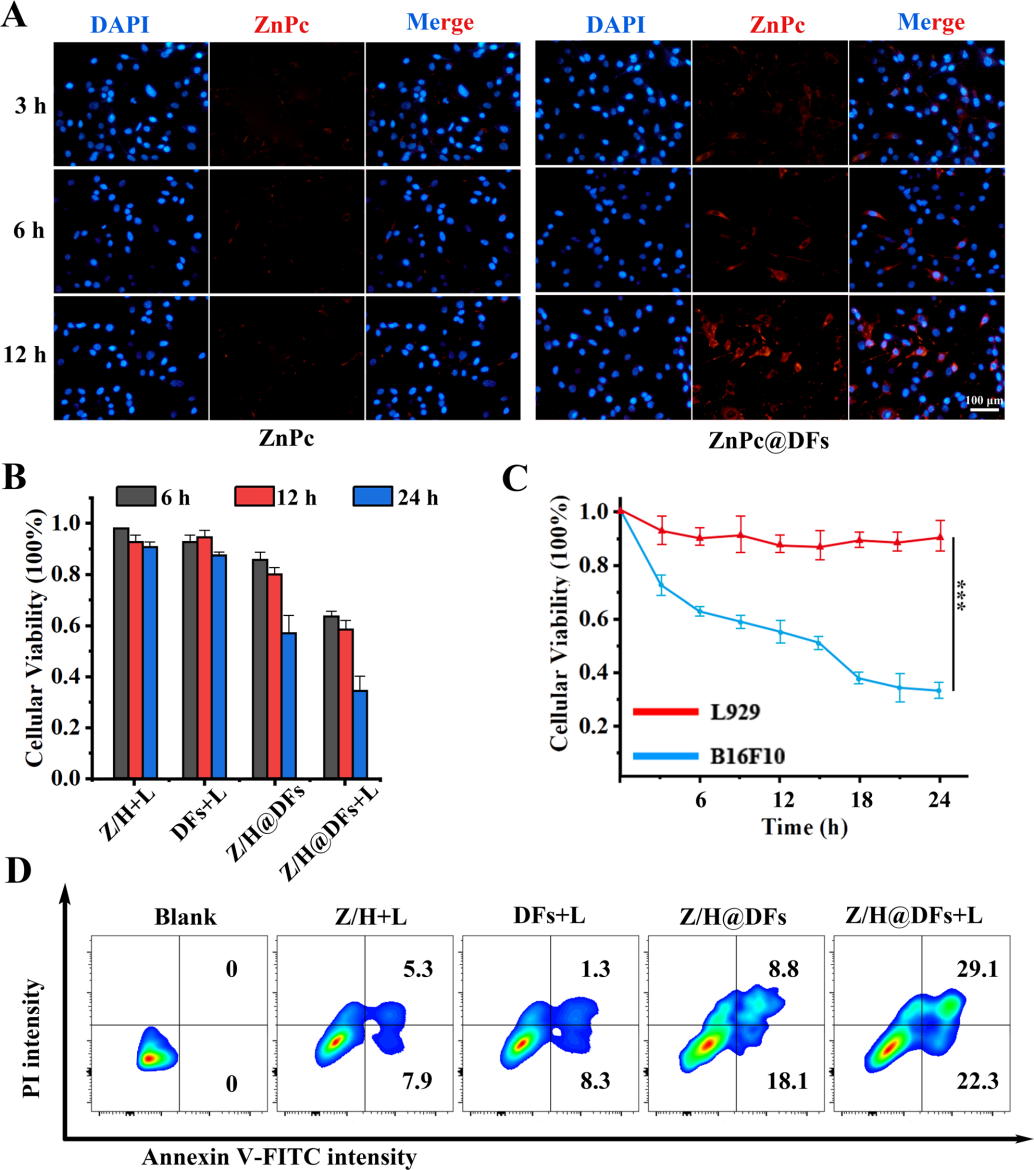
Cellular uptake and anticancer effects of Z/H@DFs **A** Fluorescence images showed that DFs increased ZnPc uptake by B16F10 tumor cells. Scale bar, 100 μm. **B** The cytotoxicity of various treatments towards B16F10 tumor cells. **C** Killing effect of DFs on L929 and B16F10. **D** Apoptosis was induced in different experimental groups: Blank, Z/H+Laser, DFs+Laser, Z/H@DFs, Z/H@DFs+Laser). Data represent mean ± SD ***p < 0.001

The cellular uptake and targeting efficiency of DFs were assessed by exposing B16F10 cells to ZnPc-loaded DFs (ZnPc@DFs) and analyzing them using fluorescence microscope and flow cytometry (Fig. 2A). After 3 h of incubation, intense fluorescence signals were detected in the ZnPc@DFs group, indicating that DFs could be specially internalized by tumor cells. Subsequently, we conducted further verification using Cy5-labeled DFs, demonstrating that loading ZnPc and Hemin had a minimal effect on targeting ablility originated from AS1411 (Fig. S2A). Furthermore, free ZnPc exhibits suboptimal biocompatibility, which is notably improved upon its association with DFs, thereby enhancing both biocompatibility and targeting of ZnPc (Fig. S2B). The anti-tumor efficacy of Z/H@DFs was investigated through various experimental analysis. The cytotoxic effects of different experimental groups were evaluated using CCK-8 assays (Fig. 2B and Fig. S3A). Z/H@DFs exhibited potent anti-tumor abilities with concentration-dependent and time-dependent effects, surpassing the efficacy of free ZnPc and Hemin. The safety of DFs was verified by means of a control cell, namely the L929 cell line. The results demonstrated that the DFs did not cause obivous harm to the normal cells (Fig. 2C).

In addition, apoptosis staining and flow cytometry analysis indicated that Z/H@DFs+Laser group exhibited the most potent apoptotic-inducing efficiency of 29.1% compared with Z/H+Laser, DFs+Laser and Z/H@DFs groups (Fig. 2D). These findings underscore DFs as superior carriers, leveraging AS1411 for mucleolin-mediated endocytosis in melanoma cells. The observed selectivity aligns with the material's biocompatibility, minimizing off-target effects—a critical advantage over conventional chemotherapeutics. Building on the oxygenation cascade, this targeted delivery potentiates PDT-induced cell death, paving the way for synergistic immunomodulatory effects.

### The evaluation of synergistic gene and photodynamic therapy effect 

**Fig. 3:**
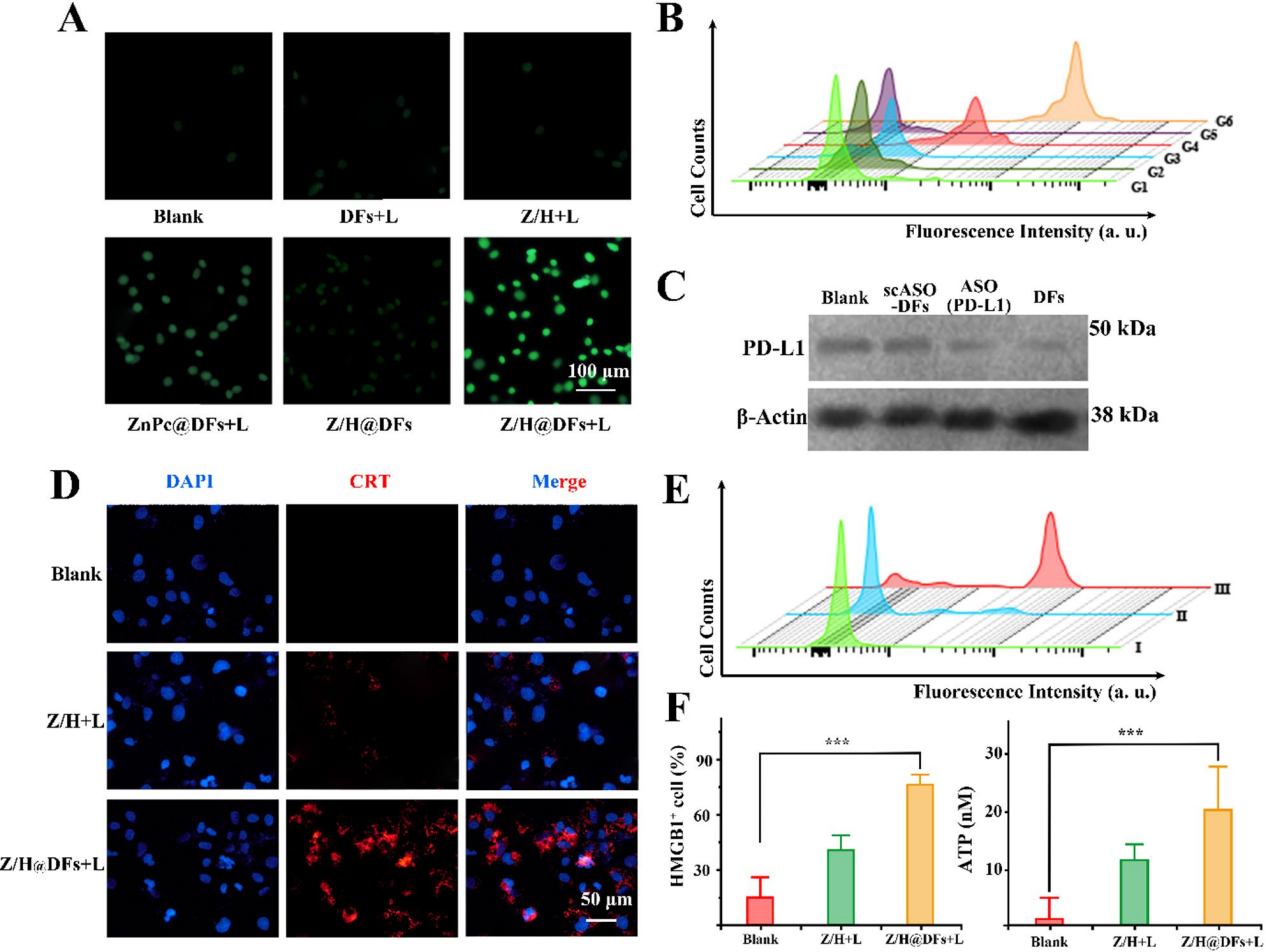
The evaluation of Synergistic gene and photodynamic therapy effect. **A** Fluorescence images showed ROS production in different experimental groups, Scale bar, 100 μm. (G1. Blank G2. DFs+Laser G3. Z/H+Laser G4. ZnPc@DFs+Laser G5. Z/H@DFs G6. Z/H@DFs+Laser). **B** Flow cytometric analysis of ROS production in different experimental groups. **C** Western blot analysis was utilized to assess the expression levels of PD-L1 among various experimental groups. **D** Fluorescence images show CRT levels in different experimental groups. Scale bar, 50 μm. (Ⅰ. Blank Ⅱ. Z/H+Laser Ⅲ. Z/H@DFs+Laser). **E** Flow analysis of different group of cells in the expression of the CRT. **F** Expression of HMGB1 and ATP in different treatment groups. Data represent mean ± SD (n = 3 biologically independent samples) ***p < 0.001

Intracellular ROS assays, including fluorescence imaging and epidermal temperature monitoring (Fig. S3A, B and S4), confirmed that Z/H@DFs with laser (Z/H@DFs+L) generated the highest ROS levels, surpassing controls like ZnPc@DFs+L or free mixtures of ZnPc and Hemin with laser (Z/H+L) (Fig. 3A and 3B). To demonstrate the impact of PD-L1 ASO on the expression of PD-L1 in tumor cells, Western blot analysis was conducted to measure the expression of PD-L1 protein in tumor cells, which demonstrated that the experimental group induced a significant downregulation of PD-L1 expression by compared to controls (Fig. 3C). Compared to free ASO, the ASO encapsulated within the DFs structure demonstrates an enhanced efficacy in reducing PD-L1 expression. This improvement can likely be attributed to the tumor-targeting capability conferred by the AS1411 component of DFs, combined with the inherent high stability of the DFs themselves   [16, 21]. Conversely, scrambled ASO-loaded DFs did not elicit similar changes in PD-L1 expression. Furthermore, we explored the immunogenicity of tumor cell apoptosis biomarkers concerning calreticulin (CRT), High-mobility group box 1 protein (HMGB1) and Adenosine 5’-triphosphate (ATP) [22]. Evaluation of CRT exposure using confocal laser scanning microscopy (Fig. 3D) and flow cytometry (Fig. 3E) revealed significantly higher CRT exposure in the experimental group compared to other groups. The elevated expression of HMGB1and ATP in the experimental group was confirmed using the ELISA assay (Fig. 3F). These results suggest that the proposed strategy have the potential to induce immunogenic cell death (ICD) in cancer cells, similar as reported in previous research [23]. This synergy stems from a core biological interplay: amplified PDT eradicates tumor cells and triggers ICD, promoting antigen presentation and immune activation. However, surviving cells often upregulate PD-L1 as a compensatory immune escape mechanism under therapeutic stress. Here, the embedded PD-L1 ASO preemptively silences this pathway, fostering a "photo-immune" cascade that amplifies anti-tumor immunity. Such integration exemplifies how gene therapy complements PDT, addressing resistance mechanisms in melanoma.

### Preparation and characterization of DFs-based microneedle

**Fig. 4:**
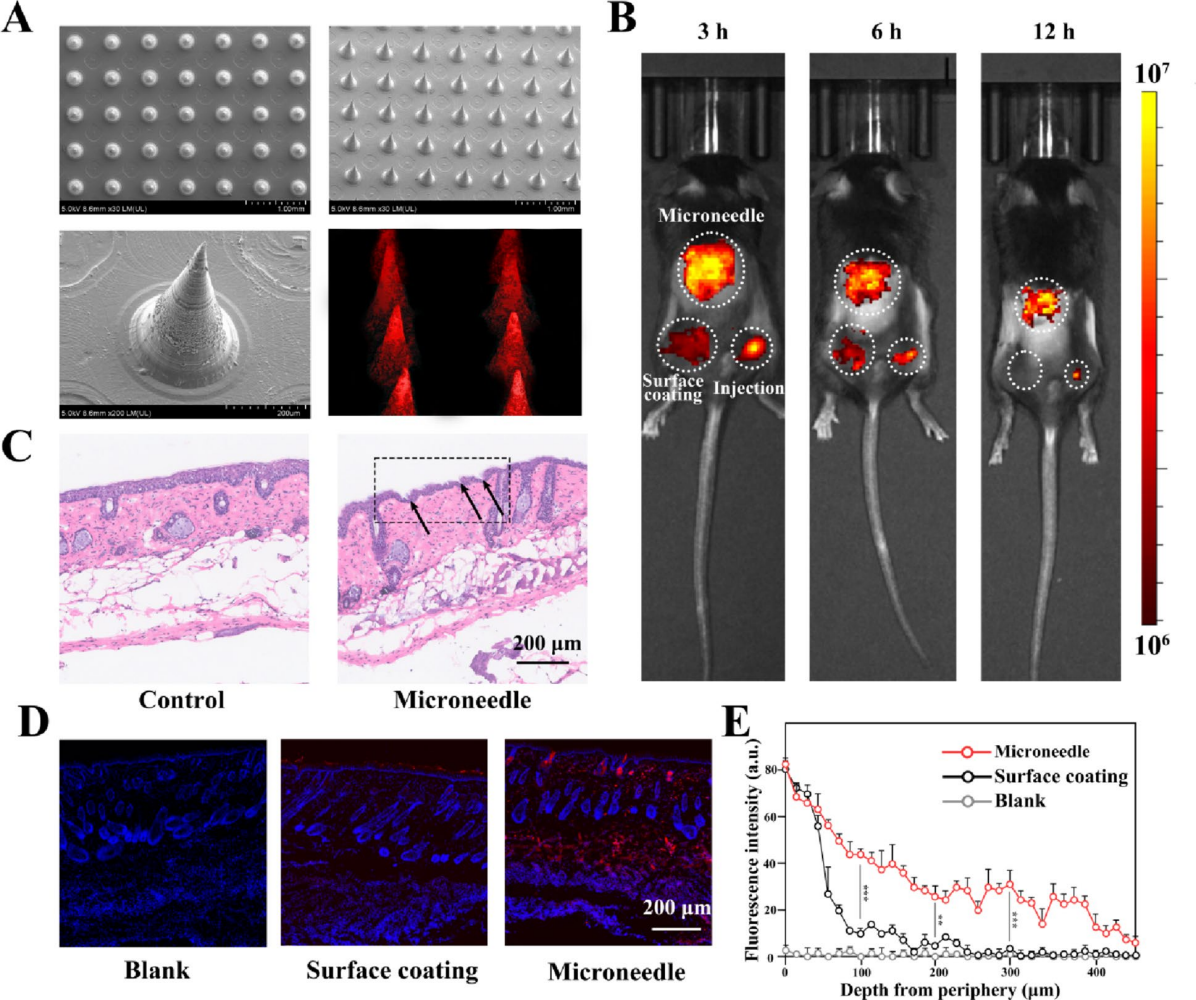
Preparation, characterization, and topical administration of MNs. **A** Microscopic image of ZnPc@DFMNs. Red fluorescence arises from the photosensiziter ZnPc. **B** The drug-releasing model operated on melanoma mice. Three approaches, including tumor injection, surface coating, and microneedle puncture, were applied to observe the time course of ZnPc diffusion in vivo. The microneedle approach provided a more effective and controlled release of ZnPc. (Refer to the Materials and Methods section for specific experimental protocols). **C** Skin tissue sections after microneedle treatment. Black arrow indicates traces after microneedling treatments. Scale bar, 200 μm. **D** Fluorescence picture of microneedle treated skin with two approaches, skin puncture and surface coating. For surface coating, HA was mixed with ZnPc@DFs and smeared uniformly across the skin surface, covering the same area as that in the microneedle group. Red fluorescence indicates ZnPc that released either from ZnPc@DFs inside the microneedle or penetrated from the skin-coated ZnPc@DFs. Scale bar, 200 μm. **E** Quantitive analysis of the released cargo (ZnPc) from the two approachs. Data represent mean ± SD, **p < 0.01, and ***p < 0.001

We incorporated DFs into a matrix of HA and fabricated DFMN patches using micro-molding techniques. The aforementioned PAGE experiments indicate that HA, when employed as a microneedle matrix, does not interfere with nucleic acid materials (Fig. 1A, S1). Furthermore, relevant studies demonstrate that high molecular weight HA can mitigate inflammation by modulating macrophage surface receptor signaling pathways, thereby safeguarding normal tissues in the presence of inflammatory conditions or tissue damage [24]. The MNs were meticulously arranged in an array, featuring a conical configuration with specific dimensions: a base diameter of 270 μm, a height of 500 μm, spacing of 700 μm and a sharp tip tapering to a radius of curvature of 5 μm. This geometric configuration not only ensures mechanical stability during the skin permeation process but also facilitates the uniform distribution of the drug by maintaining sufficient interstitial space for potential tissue deformation. This is particularly relevant for the epidermal layer and the upper dermis, where melanoma lesions are typically located. The sharp-tip geometry, characterized by a radius of curvature of 5 μm, minimizes insertion force while preserving structural integrity. Analysis of fluorescence images of the arrays revealed consistent loading of the DFs and precise alignment of the MNs, indicated by fluorescence emanating from ZnPc (Fig. 4A). Then, *in vivo* bioimaging experiments indicated that the microneedle puncture approach resulted in a more consistent and sustained release of the drug formulation compared to traditional injection and surface coating methods (Fig. 4B). We also assessed the skin penetration capability of ZnPc@DFMNs through histological examination. Skin tissue samples from the experimental group were subjected to Hematoxylin and Eosin (H.E.) staining, demonstrating that the MNs successfully penetrated the skin with minimal damage to the normal skin architecture (Fig. 4C). To highlight the advantages of the microneedle patch, we compared the microneedle puncture approach with other topical administration methods, such as surface coating and direct tumor injection. Fluorescence microscopy images illustrated that the MNs facilitated deep penetration of the ZnPc through the stratum corneum into the dermis, in contrast to surface coating (Fig. 4D). Quantitative analysis showed that the microneedle puncture approach exhibited a significantly higher ZnPc release compared to the surface coating approach (Fig. 4E). Overall, MNs strategy represents a reliable platform for synergistic photo-immunotherapy, with potential extensions to other dermatological conditions.

### In vivo validation of anti-tumor efficacy in B16F10 mouse tumor model

**Fig. 5:**
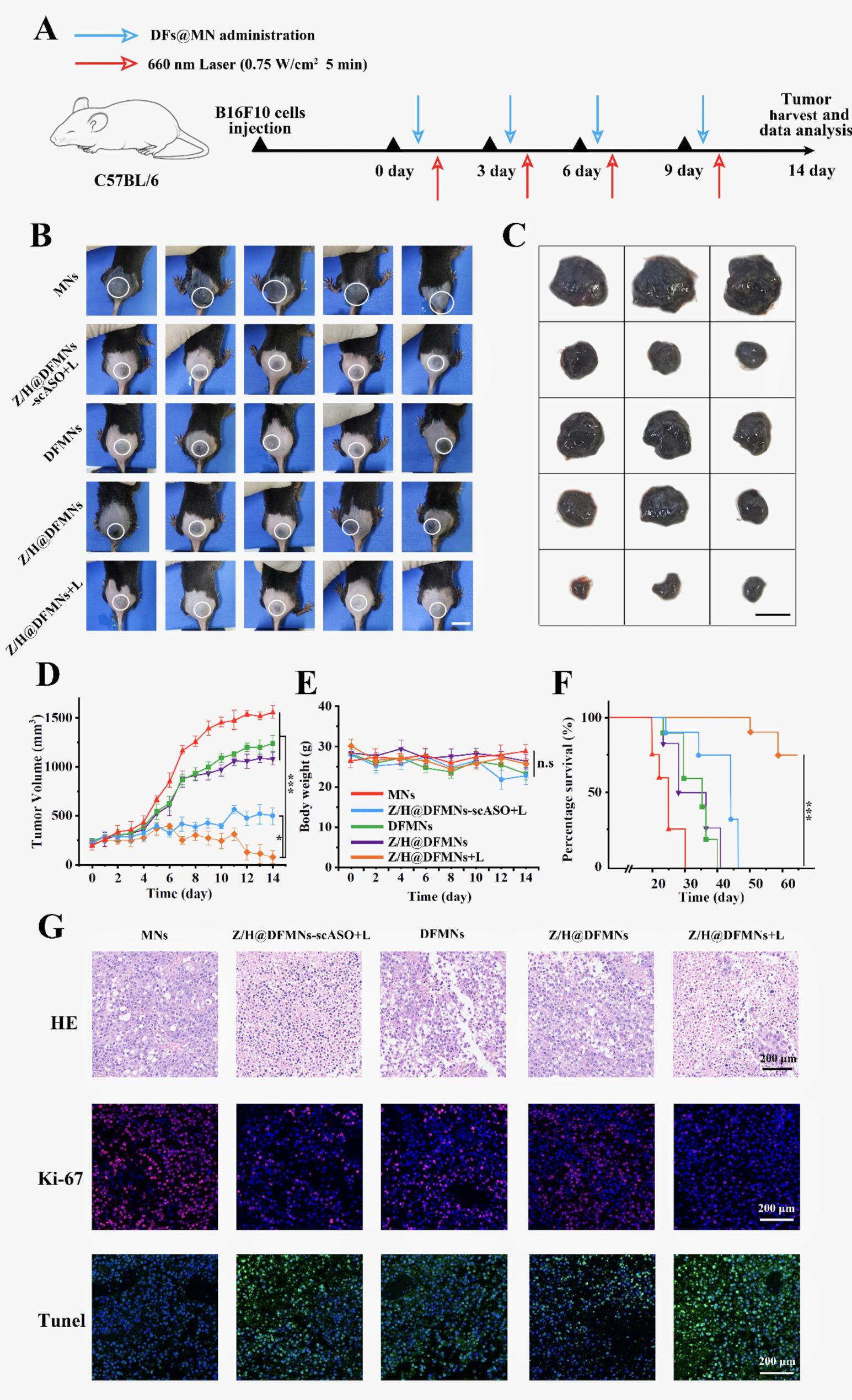
In vivo validation of anti-tumor efficacy in B16F10 mouse tumor model. **A** Schematic presentation of the treatment timeline. **B** Photo of tumor-bearing mice at day 14 after various treatments. **C** Tumor tissue obtained from the tumor-bearing Balb/c mice on day 14 after treatment. Scale bar: 1.0 cm. **D** The volume of the tumors at day 14 after various treatments. Data represent mean ± SD (n = 5 biologically independent samples). **E** The impact of different treatment regimens on body weight in mice. Data represent mean ± SD (n = 5 biologically independent samples). **F** The survival percentages of the tumor-bearing BALB/c mice (n = 5 biologically independent samples). **G** Tumor H.E. staining images, fluorescent images of TUNEL and Ki-67 expression of the tumor tissue from mice with different treatments. Scale bar, 200 μm. Data represent mean ± SD, ***p < 0.001

In this section, we conducted an investigation into the anti-tumor effects of Z/H@DFMNs *in vivo* (Fig. 5A). Tumor-bearing mice were categorized into five groups: MNs, Z/H@DFMNs-scASO + L (scASO refers to scrambled ASO sequences), DFMNs, Z/H@DFMNs, and Z/H@DFMNs + L. As shown in Fig. 5B, the Z/H@DFMNs + L group (namely the mice dosed with Z/H@DFMN and irradiated by 660 nm Laser) exhibited a significantly decreased tumor compared to other groups. The tumor growth on the mice treatment with Z/H@DFMNs but without laser irradiation was also restrained, but to a significantly less extent (Fig. 5C). However, it was noted that the DFMNs and Z/H@DFMNs group showed a similar tumor growth rate compared with the MNs control group, suggesting that PD-L1 ASO alone had a limited inhibition effect on the tumor growth. In contrast, the Z/H@DFMNs-scASO + L group exhibited moderate tumor suppression effects, indicating superior PDT efficiency via the combination of ZnPc and Hemin into the G-quadruplex structure of AS1411 aptamer. The assessment of tumor volume dynamics corroborates these findings, indicating that the Z/H@DFMNs + L group exhibited the smallest tumor volume in comparison to the other groups (Fig. 5D). Furthermore, negligible weight loss was observed across all tumor-bearing mouse groups throughout the treatment period. Comprehensive analyses of organ coefficients, blood biochemical parameters, and major organ histopathological sections revealed no significant pathological abnormalities. (Fig. 5E, Fig. S5, S6 and Table S1). Survival analysis revealed a notable survival advantage for the Z/H@DFMNs + L treated mice, with an 80% survival rate at the termination of the 60-day observational period (Fig. 5F). Noteworthy the experimental group exhibited tumor cell sparsity and detachment, accompanied by increased apoptosis (TUNEL) and reduced proliferation (Ki-67) (Fig. 5G). Overall, the Hemin/ZnPc cascade amplifies PDT by converting tumor-abundant H₂O₂ to O₂, alleviating hypoxia and boosting ROS generation, a kinetic loop that directly combats melanoma’s aggressive, oxygen-deprived microenvironment. Coupled with MNs-enabled localized delivery, this circumvents first pass metabolism, enhancing bioavailability and reducing off-target effects.

### Evaluating the activation of anti-tumor immunity

**Fig. 6:**
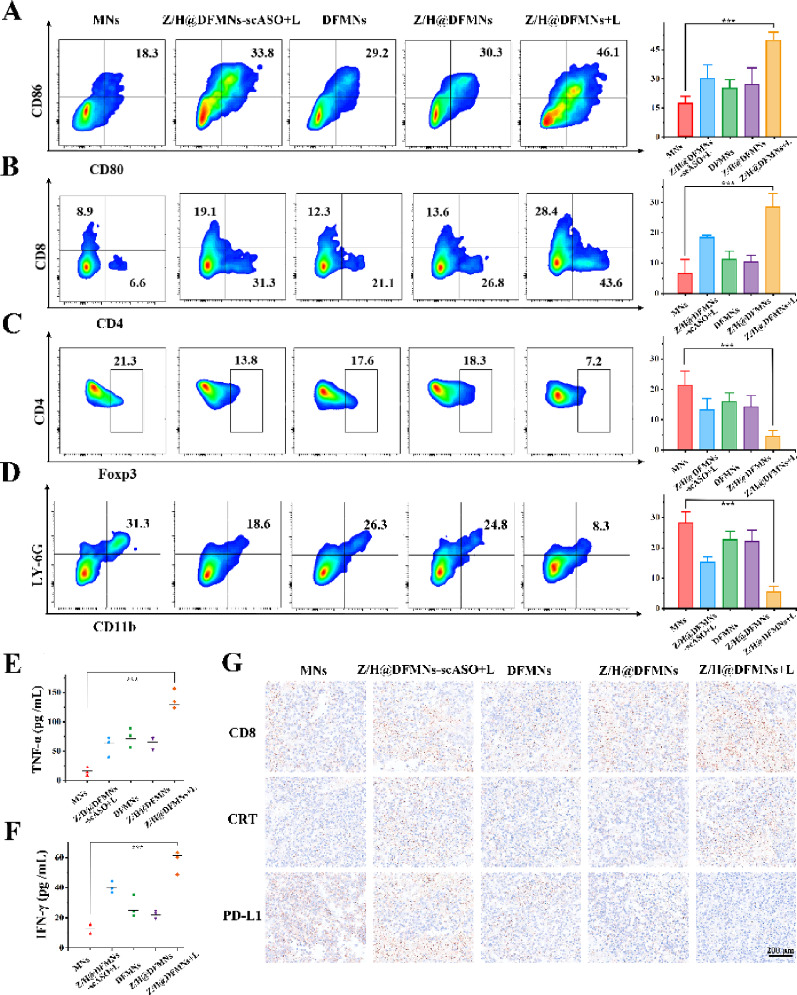
Evaluating the activation of anti-tumor immunity. **A** DC maturation induced by microneedle strategy on B16F10 tumor-bearing mice (gated on CD11c+ DC cells). Data represent mean ± SD (n = 3 biologically independent samples). **B** Flow cytometric examination of the intratumor infiltration of CD4^+^ and CD8^+^ T cells (gated on CD3^+^ T cells). Data represent mean ± SD (n = 3 biologically independent samples). **C** The Treg frequencies in tumors after different treatments. Data represent mean ± SD (n = 3 biologically independent samples). **D** Flow cytometry plots showing the frequency of MDSC in the primary tumors after different treatments. Data represent mean ± SD (n = 3 biologically independent samples). **E** and **F** TNF-α and IFN-γ levels in serum on day 14 after treatment. Data represent mean ± SD (n = 3 biologically independent samples). **G** The representative CD8, CRT, and PD-L1 stained tumor slice images after different treatments. Data represent mean ± SD, ***p < 0.001

The malignant B16F10 melanoma is a classical model in immunotherapy research due to its disinct immunological properties [25]. In order to elucidate the augmented anti-tumor immune response resulting from the synergisitic treatment of PDT and PD-L1 ASO-based gene therapy, we assessed immune cell variations in lymph nodes, tumors, and key cytokines in the serum of a subset of experimental mice (*n*=3) following a 14-day treatment period. Since mature dendritic cells (DCs) are pivotal for imitiating antigen presentation and adaptive immunity, we first evaluated their maturation status [26]. Our findings revealed that Z/H@DFMNs+L treatment elicited a significantly higher proportion of mature DCs comparted to other groups. This enhanced antigen presentation was accompanied by a robust infiltration of effector immune cells, evidenced by distinct populations of CD8^+^ cytotoxic T cells and CD4^+^ helper T cells within primary tumors (Fig. 6A, B). Crucially, to assess the remodeling of the immunosuppressive TME, we monitored the frequency of regulatory T cells (Tregs) and myeloid-derived suppressor cells (MDSCs). Notably, the Z/H@DFMNs+L group exhibited a marked reduction in Tregs (Fig. 6C) and demonstrated the lowest frequency of MDSCs (Fig. 6D) among all controls. Reflecting a systemic pro-inflammatory immune response,serum analysis showed that Z/H@DFMNs+L treatmentinduced significant upregulation of key cytokines, specifically TNF-α (Fig. 6E) and IFN-γ (Fig. 6F). Finally, to corroborate these flow cytometric findings at the tissue level, we analyzed marker proteins usingimmunohistochemistry. The immunohistochemistry images visually confirmed the triad of therapeutic mechanisms in the Z/H@DFMNs+L group: enhanced accumulation of CD8^+^ T cells (immune activation), effective downregulation of PD-L1 (gene therapy), and elevated CRT exposure (PDT-induced ICD) (Fig. 6G). It is well-established that ICD facilitates the recruitment and maturation of DCs, a critical step for priming antigen-specificadaptive immunity. Consistent with this, we observed elevated CD8⁺T cell levels in both the DFMNs (gene therapy only)and Z/H@DFMNs-scASO+L (PDT only) groups. This underscores the individualcontributions of PD-L1 blockade and PDT-mediated immune activation, respectively [27]. The Z/H@DFMNs+L group achieved the most profound effector cell infiltration, which suggests that the synergy between ASO-mediated checkpoint blockade and PDT-induced ICD maximizes the recruitment of cytotoxic T cells, overcoming the limitations of mono-therapies[28].

This immune restoration is further evidenced by a marked reduction in immunosuppressive MDSCs [29]. TNF-α and IFN-γ are crucial inflammated biomarkers existed in the TME to modulate T cell responses [30]. Additionally, the upregulated CRT expression confirms the successful induction of ICD *in vivo* [31]. Collectively, these findings demonstrate that our microneedle-mediated strategy effectively remodels the immunosuppressive TME. By bridging local intervention with anti-tumor immunity activation, this synergistic photo-immunotherapy holds great potential for effectively suppressing tumor progression and fosteringlong-term immune surveillance.

## Conclusion

In conclusion, this study presents a photo-immunotherapy platform based on DNA nanoflower-integrated microneedles.By synergizing in situ oxygen-boosted PDT with PD-L1 gene silencing, this strategy effectively overcomes the skin barrier and remediates theimmunosuppressiveTME of melanoma.Crucially,the strategy facilitate a transition from “cold” to “hot” tumors, eliciting a potent systemic immune response that inhibits both primary tumor growth and may suppress potential metastasis. Given its biocompatibility and minimally invasive nature, this system offers a promising therapeutic paradigm not only for melanoma but also for other superficial malignancies like basal cell carcinoma.Looking forward, the integration of artificial intelligence algorithms to optimize microneedle geometry and drug loading could further elevate this technology. This advancement is expected to enhance deep tissue penetration and therapeutic precision, thereby expanding its potentialfor clearing deep-seated solid tumors and metastatic foci.

## Supplementary Information

Supplementary Material

## Data Availability

The data that support the findings of this study are available in the supplementary material of this article.
