# Supplementary material for: A DNA nanoflowers-based microneedle patch for transdermal gene and photodynamic therapy against melanoma
Source: J Nanobiotechnology. 2026 Feb 27;24:311. doi: 10.1186/s12951-026-04095-w (PMC13049840; doi:10.1186/s12951-026-04095-w)
Supplement: Supplementary file 1 — Supplementary Material [file 12951_2026_4095_MOESM1_ESM.docx]

**A DNA nanoflowers-based microneedle patch for transdermal gene and photodynamic therapy against melanoma**

Yuchen Qi^1#^, Zhe Wang^1#^, Yaguang Wu^2#^, Qianqian Wu^1^, Chong Li^3^, Hua Yu^4^, Tian Zeng^1^, Yunlong Wang^1^, Jianjun Li^1^*, Hang Qian^5^*, Xiang Zhao^1^*

1. Y. Qi, Z. Wang, Q. Wu, Y. Wang, T. Zeng, Prof. J. Li, Prof. X. Zhao

Department of Oncology, Southwest Hospital, Third Military Medical University (Army Medical University), Chongqing 400038, China.

E-mail: zhaoxiang@tmmu.edu.cn, jianjunli@tmmu.edu.cn

2. Dr. Y. Wu

Department of Dermatology, Southwest Hospital, Third Military Medical University (Army Medical University), Chongqing 400038, China.

1. Prof. C. Li,

Department of Oncology,

Dazu Hospital of Chongqing Medical University, Chongqing 402360, China.

1. Dr. H. Yu,

Department of General Surgery, Hospital of Chengdu University of Traditional Chinese Medicine, Chengdu, 610072, China.

1. Prof. H. Qian

Department of Pulmonary and Critical Care Medicine, Xinqiao Hospital of Third Military Medical University (Army Medical University), 183 Xinqiao Street, Chongqing 400037, China.

E-mail: hqian@tmmu.edu.cn

**Materials and methods**

The DNA sequences (detailed sequences provided below), deoxyribonucleoside triphosphates (dNTPs), Phi29 DNA polymerase, and T4 ligase were purchased from Sangon Biotech (Shanghai, China). Hemin and ZnPc were procured from Aladdin (Shanghai, China). Hyaluronic acid methacryloyl (HAMA), polyvinyl alcohol (PVA), and the microneedle mold were purchased from the EFL brand company (Jiangsu, China). Apoptosis assay kits, CCK-8 assay kits, and 2′,7′-Dichlorofluorescin diacetate (DCFH-DA) were acquired from Beyotime Biotechnology (China). Antibodies used for flow cytometry in this study were obtained from BD Biosciences (USA). Penicillin-streptomycin solution, 0.25% (w/v) trypsin solution, and 4′,6-diamidino-2-phenylindole (DAPI) were supplied by Solarbio Co., Ltd (Beijing, China). Dulbecco's modified Eagle's medium (DMEM) and fetal bovine serum (FBS) were sourced from Corning (USA).

**Cell culture**

L929 (mouse fibroblast cell line) and B16F10 cells (mouse melanoma cell line) were selected for cell experiments. B16F10 cells were seeded in RPMI 1640 medium supplemented with 10% FBS, penicillin (100 units mL^−1^) and streptomycin (100 μg mL^−1^) in 5% CO_2_ at 37 ℃. L929 cells were seeded in DMEM medium supplemented with 10% FBS, penicillin (100 units mL^−1^) and streptomycin (100 μg mL^−1^) in 5% CO_2_ at 37 ℃.

**Preparation of DFs**

The experimental protocol by Yang et al (*Nat. Protoc.* 16, 5460-5483 (2021)). was consulted for the synthesis of DNA nanoflowers (DFs). In brief, the process involved mixing T4 ligase buffer, template, and primers, followed by incubation at 95 °C for 10 minutes, then cooling to room temperature. Subsequent addition of T4 ligase and incubation at 25 °C for 4 hours facilitated the formation of the circular template. The circular template (50 nM), along with dNTP (1 mM), BSA (0.2 mg mL^−1^), phi29 polymerase (0.0015 U L^−1^), and phi29 polymerase buffer (50 mM Tris-HCl, 10 mM MgCl_2_, 10 mM (NH4)_2_SO_4_, 4 mM DTT, pH 7.5), was gently mixed in ice bath and incubated for 2 hours at 37 °C, followed by a 10-minute incubation at 65 °C. After cooling to room temperature, DFs were harvested through centrifugation at 20,000 rpm for 20 minutes, and then incubated in a buffer (20 mM Tris, 40 mM NaCl, 40 mM KCl, pH 7.6) at room temperature for 1 hour to form G4 structures. Subsequently, the DFs were incubated with ZnPc and Hemin both pre-dispersed in DMSO (ZnPc 50 μM/ Hemin 40μM) and stirred for 2 hours under light protection. Subsequently, the obtained mixture was placed in a centrifugal filtration device with a molecular weight cutoff (MWCO) of 100 kDa to remove unloaded small molecules (ZnPc and Hemin). Afterwards, it was washed twice with buffer solution to obtain purified Z/H@DFs. The nanoparticles were characterized using various techniques including dynamic light scattering (DLS), element mapping images, transmission electron microscopy (TEM), and ultraviolet spectrophotometry (UV).

**Synthesis and stability of DFs analyzed by gel electrophoresis**

Polyacrylamide gel electrophoresis (PAGE) was used to evaluate the synthesis efficiency and stability of DFs. The DFs was introduced into DMEM supplemented with 10% FBS, followed by incubation at 37°C for 24 hours to assess its resistance to enzymatic degradation. Additionally, to further validate biological stability in a rodent-specific context, DFs were incubated in mouse serum at 37°C for 24 hours. For storage stability evaluation, the samples were maintained at room temperature in HA matrix for 72 hours. Stability validation was conducted through these standardized protocols.

**In vitro analysis of O_2_ production and Singlet oxygen generation**

The catalytic oxygen production capacity of DFs was assessed utilizing a portable dissolved oxygen meter. Samples from various treatment groups were introduced into a solution containing 10 mM H_2_O_2_, and alterations in the concentration of dissolved oxygen were monitored in real-time. A control group consisting of phosphate-buffered saline (PBS) with a pH of 7.4 and 10 mM H_2_O_2_ was employed for comparative purposes. The Singlet Oxygen Sensor Green (SOSG) was utilized as a reliable indicator of singlet oxygen production in DFs following laser irradiation. DFs were incorporated into a solution containing SOSG (2.5 μM), and distinct samples were subjected to light irradiation at a wavelength of 660 nm and intensity of 0.75 W cm^-^². The fluorescence intensity of SOSG was quantified using a fluorescence spectrophotometer (Molecular Devices, USA) (Ex = 490 nm, Em = 525 nm). To explore the impact of external variables on ROS production by DFs, the samples were pre-exposed to 10% FBS for a duration of 12 hours.

**Cellular uptake study**

To prepare Cy5-labeled DFs for cellular uptake study, Cy5-dCTP was incorporated into the RCA reaction mixture. B16F10 cells were cultured overnight in wells with a concentration of 3×10^5^ cells per well. DFs, DFs-scAS1411, Z/H@DFs, ZnPc@DFs, and ZnPc were treated at the same concentrations of DFs and/or ZnPc. After removing the supernatant, the cells were fixed with paraformaldehyde, stained with DAPI for nuclear visualization, and sealed with glycerol. The cellular uptake efficiency was then qualitatively assessed under fluorescence microscope (Olympus, Germany). Furthermore, the fluorescence intensity within the cells was quantitatively measured by harvesting and analyzing the cells using flow cytometry (FACSVerse, BD, USA).

**In vitro cytotoxicity study**

The cytotoxic effects of DFs on B16F10 cells were evaluated through in vitro experiments utilizing the CCK-8 assay. B16F10 cells were seeded in 96-well plates and subsequently exposed to various treatments in fresh medium: G1. Blank, G2. ZnPc/Hemin+Laser (Z/H+L) (Z/H was prepared by free ZnPc with Hemin mixture), G3. DFs+Laser (DFs+L), G4. ZnPc/Hemin@DFs (Z/H@DFs), G5. ZnPc/Hemin@DFs+Laser (Z/H@DFs+L). Following a 12-hour incubation period, the cells were exposed to a 660 nm laser at 0.75 W cm^-2^ for 1 minute. After 24 hours, 10 μL of CCK-8 reagent was added to each well and incubated for 1 hour. The absorbance at 450 nm was measured. Additionally, cytotoxicity assessments were conducted through flow cytometry analysis using the Annexin V-FITC Apoptosis Detection Kit (Beyotime, China).

**Intracellular ROS generation**

Intracellular levels of reactive oxygen species (ROS) in B16F10 cells following various treatments were assessed utilizing the DCFH-DA fluorescent probe. Initially, B16F10 cells were seeded in 12-well plates. Subsequently, the ROS inhibitor NaN_3_ was introduced to the cell suspension and incubated for 1 hours to scavenge ^1^O_2_ within the B16F10 cells. Next, PBS, DFs, ZnPc@DFs, Z/H, and Z/H@DFs (ZnPc 7.5 μM) were added to 1mL of the culture medium. Following a 12-hour incubation period, the cells were rinsed with PBS and treated with DCFH-DA (25 μM) for 20 minutes. Subsequent to this, cells were exposed to irradiation at 660 nm (0.75 W cm⁻²) for 1 minute as per experimental group requirements. Fluorescence images were then observed using an inverted fluorescence microscope, followed by quantitative analysis of fluorescence intensity via flow cytometry.

**Western blot analysis of PD-L1 protein**

B16F10 cells were seeded at a density of 4×10^5^ cells in a 6-well plate and subjected to various treatments for a duration of 48 hours. Subsequently, the cells were lysed using RIPA buffer to extract total cellular proteins. The concentration of proteins was quantified using a BCA protein detection kit (Beyotime, China). Western blot analysis was conducted following standard procedures. Briefly, the proteins were separated by SDS polyacrylamide gel electrophoresis (SDS-PAGE), transferred onto PVDF membranes (Bio-Rad, USA), and then incubated overnight at 4 °C with specific primary antibodies. Detection of target proteins was achieved using HRP-conjugated secondary antibodies. Visualization of protein bands was accomplished by exposing the membranes to an ECL chemiluminescent substrate and imaging with the ChemiDoc MP system (Bio-Rad, USA).

**Preparation and characterization of microneedle**

In this study, drug-loaded microneedles (DFMNs) were prepared according to a specific protocol. Initially, drug formulations (DFs) were combined with 5% (w/v) hyaluronic acid methylcellulose (HAMA) and introduced into polydimethylsiloxane (PDMS) microneedle master structures. Centrifugation was then used to eliminate any air bubbles. The filled molds underwent a drying process at 45 °C, followed by an additional drying step involving the incorporation of a 20% (w/v) polyvinyl alcohol (PVA) solution to create the microneedle substrate. Subsequently, the DFMNs were extracted from the PDMS molds to yield the final product. The morphological characteristics of the DFMNs were analyzed using scanning electron microscopy (SEM) and fluorescence microscope (Olympus, Germany). Furthermore, Cy5-labeled DFs were loaded into the microneedles and allowed to incubate for 12 hours post-treatment. Histological sections of mouse skin were then examined using fluorescence imaging and H.E. staining techniques.

**In vivo imaging**

To explore the impacts of diverse delivery modalities, B16F10 tumor-bearing mice were exposed to intratumoral injection, microneedle puncture, and surface coating (All treatments utilized an equal amount of ZnPc. For surface coating, hyaluronic acid was combined with ZnPc@DFs and smeared uniformly across the skin surface, covering the same area as that in the microneedle group for durations of 3, 6, and 12 hours. Following treatment, the mice were subjected to the IVIS Lumina XRMS Series III system.

**In vivo antitumor efficacy**

The antitumor efficacy of DFMNs was investigated in vivo using B16F10 tumor-bearing mice. The mice were randomly assigned to five groups, each consisting of five mice: MNs; Z/H@DFMNs-scASO with laser treatment; DFMNs; Z/H@DFMNs; and Z/H@DFMNs with laser treatment. MNs was percutaneously inserted into the tumor site at a dosage of 5 mg kg^-1^, calculated based on ZnPc. Laser treatment with a 660 nm wavelength at 0.75 W cm^-2^ for 5 minutes was administered to the laser groups. The specified treatments were applied on days 1, 4, 7, and 10. On day 14, mice were euthanized to collect serum for biochemical analysis (ALT, AST, BUN, CRE) and major organs for H.E. staining. To further evaluate long-term systemic biosafety, organ coefficients were calculated on day 14 and in a separate cohort on day 28, respectively.

**Cytokine detection**

Serum samples were collected from mice on the fourteenth day following different treatments and were subsequently diluted for analysis. The concentrations of TNF-α and IFN-γ were determined using enzyme-linked immunosorbent assay (ELISA) kits in accordance with the manufacturer's protocols (Elabscience, China).

**Immune cell analysis**

To evaluate the systemic and local immune responses, single-cell suspensions were isolated from excised primary tumors, and tumor-draining lymph nodes. For phenotypic analysis of immune infiltrates, cells were stained with fluorochrome-conjugated antibodies according to standard protocols.

Specifically, T cell populations were characterized using anti-CD45-PE-Cy7, anti-CD3-FITC, anti-CD4-BV421, and anti-CD8-PerCP-Cy5.5. Conventional dendritic cell maturation was assessed by staining with anti-CD45-PE-Cy7, anti-CD11c-BV510, anti-CD80-APC, and anti-CD86-PE. Granulocytic myeloid derived suppressor cells were identified using anti-CD11b-APC and anti-LY-6G-PE.

For regulatory T cell analysis, cells were first surface-stained for CD3, CD4, and CD45, followed by fixation and permeabilization to facilitate intracellular staining of Foxp3 using an anti-Foxp3-PE antibody. All flow cytometry data were acquired and analyzed to quantify the proportions of respective immune cell subsets. All antibodies were purchased from BD Biosciences (USA).

**Histological analysis**

On the 14^th^ day of the experiment, all mice were euthanized, and tumors were harvested for histopathological and immunohistochemical analysis. The tumor sections were investigated by H.E. staining, TUNEL staining, Ki-67 and immunohistochemical staining for PD-L1, CRT, and CD8 markers. Additionally, major organs were collected for H.E. staining to assess the safety profile of the proposed strategy used in the study.

**Statistical analysis**

Experiments were conducted a minimum of three times, and the results are presented as means ± standard deviation (SD). Statistical comparisons were performed using two-tailed unpaired Student’s t-tests for two-group analyses or one-way analysis of variance (ANOVA) followed by Tukey's post hoc test for multiple-group comparisons in GraphPad Prism 10.0. A *p*-value of < 0.05 was considered statistically significant. Significance levels are indicated as **p* < 0.05, ***p* < 0.01, and ****p* < 0.001, while non-significant differences are marked as “n.s.”.

**Supplementary Figures**

**
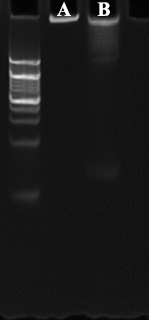
**

**Fig.S1**: PAGE analysis of DFs in HA for 72 hours (A) and in mouse serum for 24 hours(B).


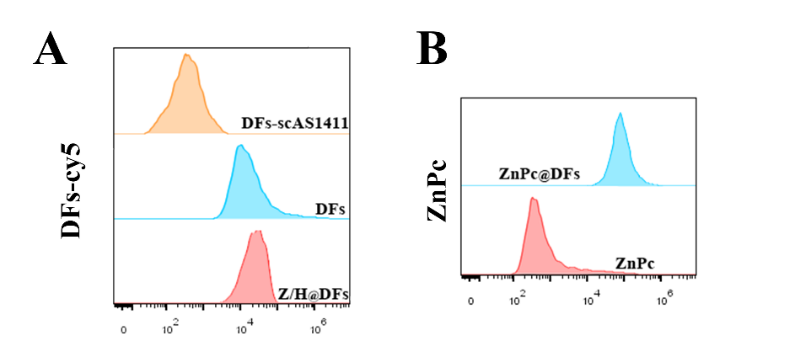


**Fig.S2 A**. Flow cytometric analysis of cellular uptake efficiency (12 h) of different DFs. **B**. Flow cytometric analysis of cellular uptake efficiency (12 h) of ZnPc under different strategies.

**
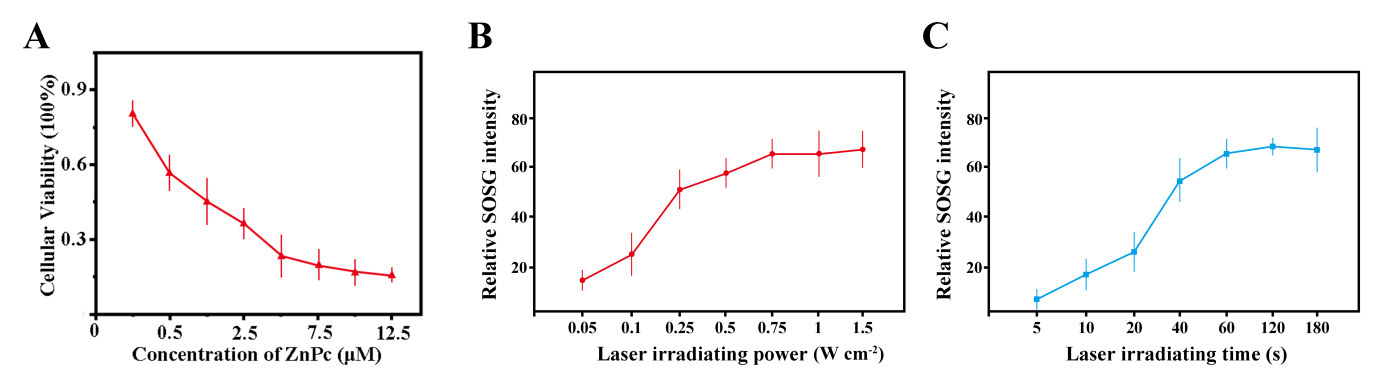
**

**Fig.S3 A**. The cytotoxic effects of different concentrations of DF (with ZnPc concentrations of 0.25-12.5 μM) under conditions of laser at 0.75 W cm^-2^ for 1 minute. **B**. Relative SOSG fluorescence intensity (indicating singlet oxygen generation) in B16F10 cells treated with ZnPc@DFs (0.75 μM ZnPc) under 660 nm laser irradiation at different power levels (0.1–0.75 W cm⁻²). **C**. Relative SOSG fluorescence intensity in B16F10 cells treated with ZnPc@DFs under 660 nm laser irradiation (0.75 W cm⁻²) for varying durations (0–180 s). Data are presented as mean ± standard deviation (n = 3 independent experiments).


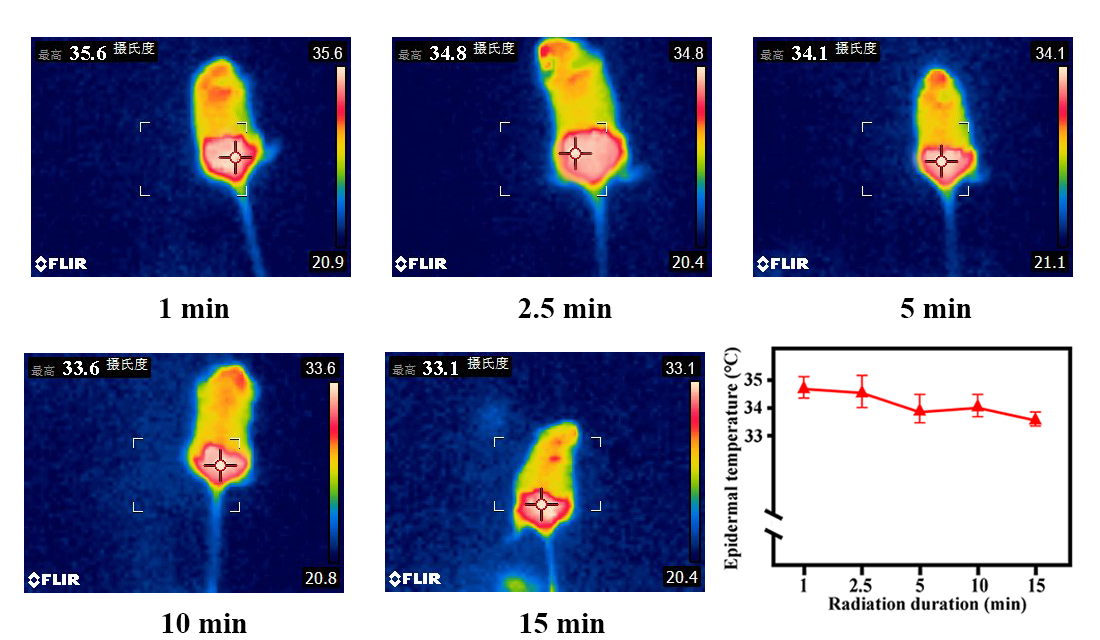


**Fig.S4:** Under conditions of constant total energy from laser irradiation, thermal imaging was utilized to record the effects of varying exposure durations on the epidermal temperature of mice.


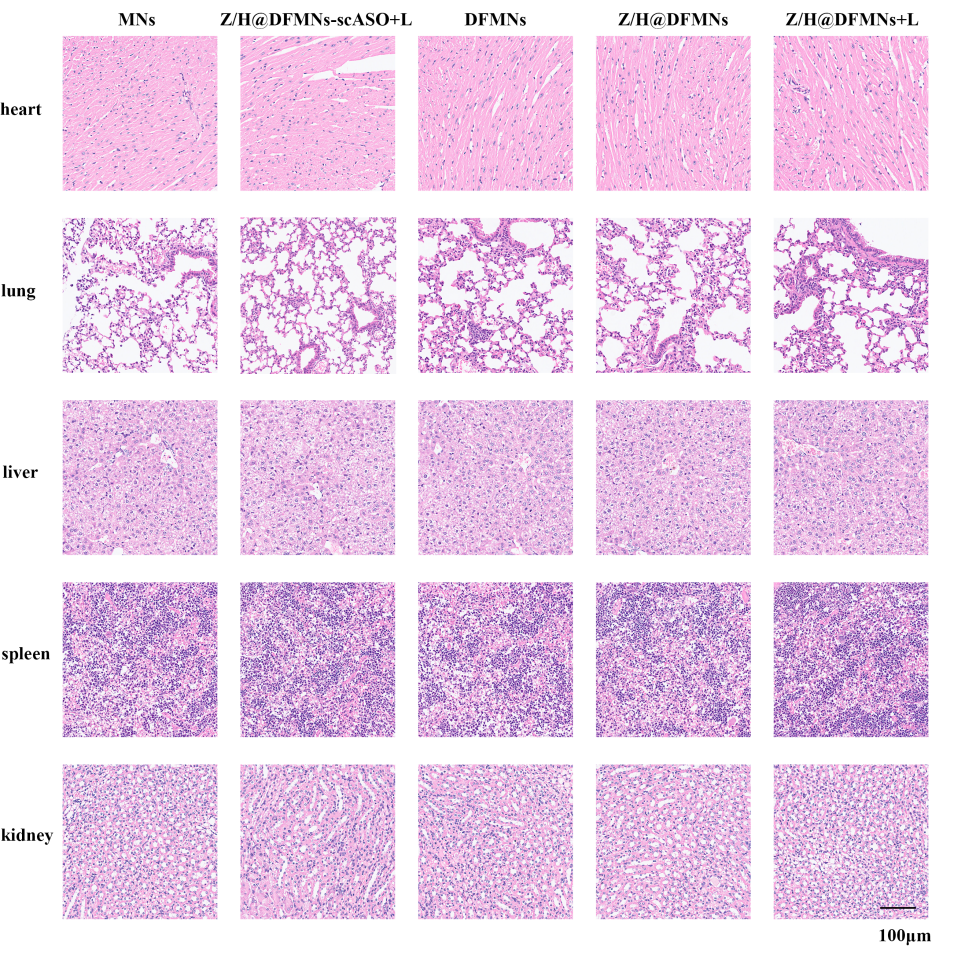


**Fig.S5** The representative H.E. staining assay of excised major organs after different treatments at day 14. Scale bar is 100 μm.


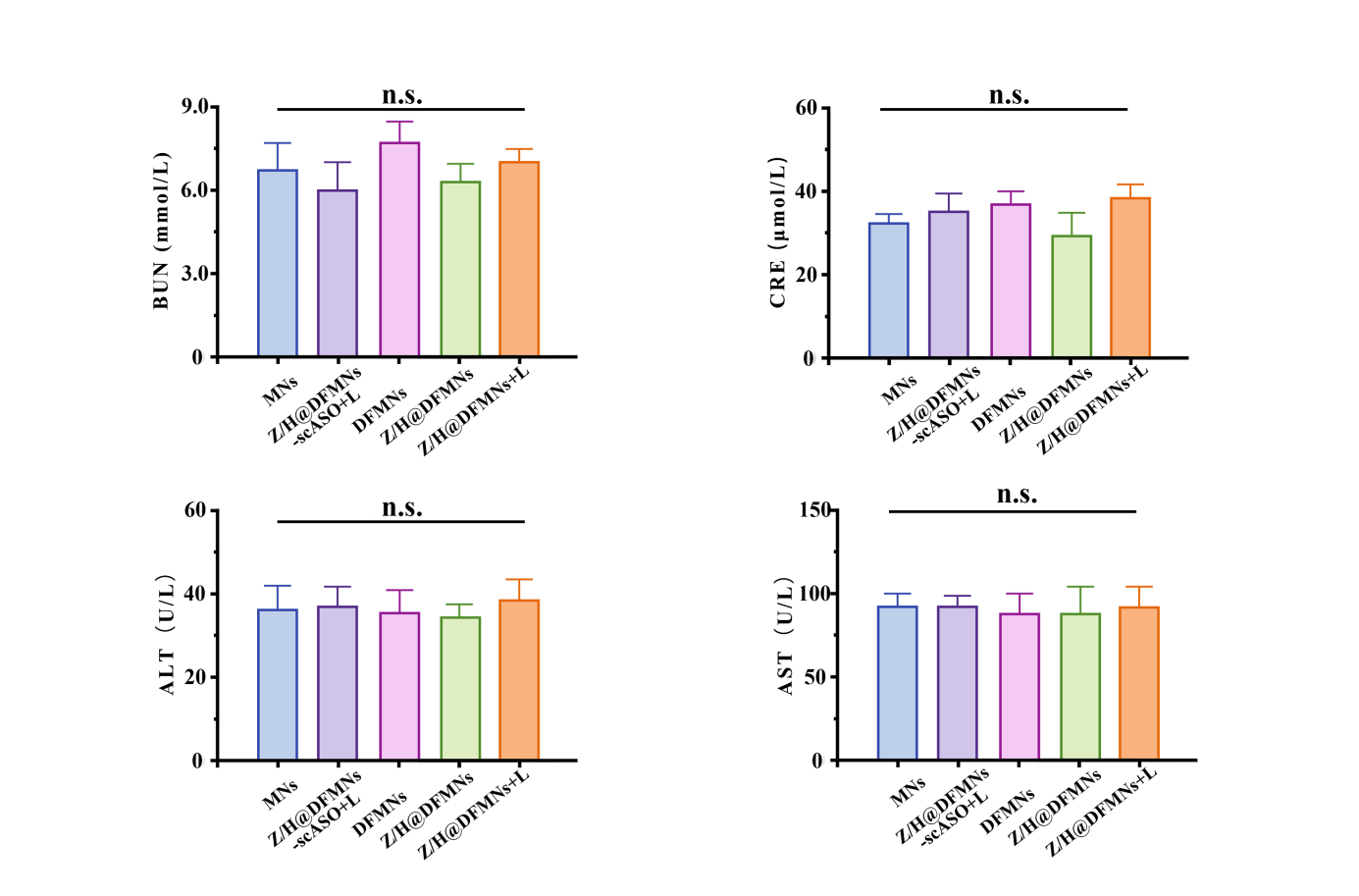


**Fig.S6：**Serum levels of ALT, AST, BUN, and CRE in mice on day 14 after treatment (n = 5 biologically independent samples)

**Supplementary table**

| **Group** | **Liver(%)** | **spleen(%)** | **kidney(%)** |
| --- | --- | --- | --- |
| **control** | 3.9±0.3 | 0.31±0.1 | 0.77±0.2 |
| **14d** | 3.7±0.6 | 0.29±0.08 | 0.83±0.1 |
| **28d** | 4.3±0.4 | 0.32±0.07 | 0.88±0.13 |

Table S1: Organ coefficient statistics of mice on the 14th and 28th days after treatment (n = 5 biologically independent samples)

| Name Sequences (5'-3') | |
| --- | --- |
| Primer | TGGTGGTGGTTGTGGTGGTGGT |
| Template | Pho-AACCACCACCACCAAGACGAGAATGCAGAGGAGCTAAGACGACAGCACAGCAACTTCAGGGAAGACGAGAATGCAGAGGAGCTAAGACGACCACCACCACCAC |
| scASO-Template | Pho-AACCACCACCACCAAGACGAGAATGCAGAGGAGCTAAGACGAAGTCGCCCTCTAAGGTTACAAGACGAGAATGCAGAGGAGCTAAGACGACCACCACCACCAC |
| Primer -2 | TAATAATAATTGTAATAATAAT |
| scAS1411- Template | Pho-AATTATTATTATTAAGACGAGAATGCAGAGGAGCTAAGACGACAGCACAGCAACTTCAGGGAAGACGAGAATGCAGAGGAGCTAAGACGATTATTATTATTAC |
| ASO  (PD-L1) | CCCTGAAGTTGCTGTGCTG |

The information of the DNA sequence used in the experiment.

Note: The PD-L1 ASO sequence was synthesized with a fully phosphorothioated backbone to ensure stability.
